# Alveolar macrophage modulation via the gut–lung axis in lung diseases

**DOI:** 10.3389/fimmu.2023.1279677

**Published:** 2023-11-21

**Authors:** Zijian Chen, Yangqi Liu, Weizhe Huang

**Affiliations:** Department of Cardiothoracic Surgery, The Second Affiliated Hospital of Shantou University Medical College, Shantou, China

**Keywords:** alveolar macrophages, gut-lung axis, dietary, microbiota, lung diseases, immunity

## Abstract

Several studies have demonstrated great potential implications for the gut–lung axis in lung disease etiology and treatment. The gut environment can be influenced by diet, metabolites, microbiotal composition, primary diseases, and medical interventions. These changes modulate the functions of alveolar macrophages (AMs) to shape the pulmonary immune response, which greatly impacts lung health. The immune modulation of AMs is implicated in the pathogenesis of various lung diseases. However, the mechanism of the gut–lung axis in lung diseases has not yet been determined. This mini-review aimed to shed light on the critical nature of communication between the gut and AMs during the development of pulmonary infection, injury, allergy, and malignancy. A better understanding of their crosstalk may provide new insights into future therapeutic strategies targeting the gut–AM interaction.

## 1 Introduction

The gut–lung axis is a highly complex pathway. During the occurrence and development of lung diseases, a normal gut environment or dietary patterns helps build the necessary pulmonary immunity, and metabolites are directly or indirectly involved in immune modulation. Conversely, gut dysbiosis and inappropriate treatment usually induce aggravation (1–3). However, the cellular mechanism behind the gut–lung axis is unclear. It is well-known that alveolar macrophages (AMs), being highly specialized tissue-resident macrophages, are the key players in innate immunity and maintaining lung homeostasis; moreover, their functions change profoundly in various lung diseases. The origin, phenotype, and function of AMs and their role in phagocytosis and immune response have recently been elucidated (4, 5). This mini-review aimed to reveal the role of AMs in gut-lung interactions. The structural, metabolic, and cellular changes in this “target cell” mediated by the gut may help further understand the pathophysiology of lung diseases, derive new insight to improve the efficacy of some current treatments, and develop novel therapies based on the gut–lung axis concept.

## 2 AMs and lung diseases: modulation based on gut–lung axis mechanisms

### 2.1 Pulmonary infection

In the past few years, extensive studies have examined the role of the gut–lung axis in bacterial and viral infections. We hereby review how gut–lung axis-mediated AM modulation shapes the host immune defense against pulmonary infections as well as the crosstalk between this pathway and current therapies.

#### 2.1.1 Bacterial pneumonia

Pneumonia causes more deaths than any other infectious diseases worldwide, especially since the coronavirus disease 2019 (COVID-19) pandemic (6). *Streptococcus pneumoniae* is the most common cause of community-acquired pneumonia (7). The gut microbiota (GM) contains pathogen-associated molecular patterns, as regulators of innate immunity, it modulates the metabolic pathways of AMs, and its depletion would weaken AM responsiveness toward bacterial virulence factors (8). Acetate, a metabolite produced by GM, enhances the phagocytic activity of AMs against *S. pneumoniae* in a nitric oxide (NO)-dependent manner (9). Even in the face of the more lethal *K. pneumoniae* pneumonia, short-chain fatty acids (SCFAs) can promote AMs to eliminate *K. pneumoniae* through LAMtor2-dependent signaling pathways (10). It is important to note that, although *K. pneumoniae* infection causes profound changes in GM, supplementation with multiple SCFAs can salvage impaired AM function and improve outcomes (11). In addition, for pneumonia caused by *Streptococcus suis* and melioidosis, GM has a positive role in promoting AM-mediated immune responses (12, 13). Overall, necessary GM and its metabolites affect lung subjects to infection, showing protective effects by arming AMs. Moreover, colonization of *S. pneumoniae* in human nasopharynx has a positive effect in enhancing AMs responsiveness to bacterial pathogens. This not only involves aspirated *S. pneumoniae* as an immune stimulus, but may also involve indirect stimulation from systemic reactions, the so-called gut-lung axis (14).

Although the discovery of antituberculosis therapy (ATT) that kills *Mycobacterium tuberculosis* (Mtb) brings hope to patients with tuberculosis, an increasing susceptibility of individuals who received medication toward reinfection or reactivation of Mtb raises doubts about whether widely used ATT has an off-target effect (15). A recent study reported that prior to Mtb infection, the use of isoniazid and pyrazinamide, rather than rifampicin, has a unique impact on GM, which in turn prevents the development of pulmonary immunity against Mtb (16). This is probably because of the impaired metabolism of AMs; an internal cellular environment that allows Mtb to grow is formed (17). Moreover, vaccination is an effective way to stop tuberculosis from spreading and the important role of AMs has been revealed, but the mechanism remains unclearly understood (18). A study has revealed a novel method by which subcutaneous Bacille Calmette–Guérin vaccination triggers immune alerts and forms immune memory of AMs in distal lung tissues, surprisingly this process is driven by changes in GM, and the induction of memory AMs is independent of circulating monocytes (19). This finding is contrary to the widely held view that the generation of immune memory is divided according to pathogen exposure site (20). However, this GM-mediated pathway will enhance our understanding of host defense mechanisms in distal organs. Notably, most of the current human vaccines are inoculated through the skin, including the COVID-19 vaccine (21).

*Pseudomonas aeruginosa* pneumonia is severe and fatal infection in hospitalized patients, especially those in intensive care units. Their prognosis depends on the use of antibiotics (Abx) as well as the pathogen’s virulence and the host’s immune response (22). *P. aeruginosa* ventilator-associated pneumonia mainly occurs in critically ill patients receiving overtime mechanical ventilation (MV) (23). Prolonged Abx use is usually necessary to reduce the recurrence rate; on the other hand, Abx treatment reduces the expression of Reg3β and TLR4 in the gut, which reduces the phagocytic activity of AMs against *P. aeruginosa* (24, 25). It should be noted that MV itself also reduces the pulmonary defense by increasing reactive oxygen species production of AMs; thus, the early intravascular injection of SCFAs, a derivative of GM, based on the gut–lung axis may help offset the negative effects (26). Unnecessary, prolonged, or broad-spectrum Abx exposure will induce intestinal barrier dysfunction, thereby weakening the phagocytosis of AMs. The oral supplementation of dead bacteria is conducive to reversing the compromised lung defense (27). Although probiotics may be more effective at activating the phagocytic capacity of AMs compared to dead bacteria, Abx treatment may inevitably affect the role of probiotics, and further research is needed to verify their relationship.

Pulmonary infections are more deadly for some high-risk people. Experiencing major surgery with severe tissue trauma tends to be immunosuppressive, especially for critically ill patients. This adverse effect can be improved by dexmedetomidine, which regulates GM to rescue the impaired phagocytic activity of AMs and reduce M2 polarization of macrophages (28). Moreover, influenza can induce GM disturbance, thereby reducing SCFA production and weakening the bactericidal activity of AMs, which is more likely to cause concurrent bacterial pneumonia. Such a superinfection could further lead to excessive mortality of influenza in these patients (29). Pulmonary infections are always fatal for patients with advanced HIV infection and concomitant GM and macrophage dysfunction. The alveolar mucosa, an important HIV pool, was a previously ignored mediator for continuous pneumonia (30, 31). However, the role of AMs in HIV–pneumonia patients remains unclear. Despite heterogeneity, many concurrent local or systemic diseases tend to impair lung defense function by affecting the gut environment, and an increased understanding of the gut–AM axis may have broader implications.

Neonatal pneumonia is the most common cause of infection leading to infant death. Although perinatal Abx use is crucial, it may increase the likelihood of life-threatening pneumonia later in infancy (32, 33). This is probably due to AM dysfunction caused by destruction of the GM balance. Interestingly, despite later fecal microbiota transplantation, some of the immune functions associated with chemotaxis and tissue repair could not be restored (34). In early life, to prepare for initial environmental exposure, macrophage precursors occupy the alveolar niche and become tissue-resident AMs (TRAMs) (35). Therefore, we must verify whether GM dysregulation impacts the developmental trajectory of AMs. Moreover, perinatal feeding practices and the delivery mode also greatly influence the neonatal GM; further research is needed to describe their role in AM origin (36, 37).

Previous studies have shown that the enhanced bacterial killing activity of AMs is mediated by Toll-like receptor (TLR) signals triggered by GM. When GM becomes depleted, the oral administration of lipopolysaccharide can help restore the immune function of AMs (38, 39). Interestingly, another study indicated that it was not the TLR, but Nod-like receptor (NLR) ligands, could rescue early bacterial clearance activity in lung (40). The reason of above contradict results is unknown, it may be because compared to NLR, TLR activation is more tightly restrained, so the effect of triggering the immune response to infection in the early stage is not as good as NLR (41). However, TLR and NLR pathways in the gut is complex, more experiments are needed to elucidate their role, understanding their mechanism facilitates more precise treatment.

#### 2.1.2 Viral pneumonia

Previous studies have provided evidence of the immunomodulatory potential of AMs in viral infections (42). AM origin is a determinant in shaping extended function against pulmonary viral infections and affecting infection severity (43). In addition to necessary antiviral responses, tissue damage during infection (such as influenza and COVID-19) caused by exaggerated immune inflammation greatly contributes to the high mortality rates (44). Surprisingly, by inducing changes in bone marrow hematopoietic function, a high-fiber diet (HFD) prevents severe influenza infection by reducing tissue damage and enhancing adaptive antiviral immunity while significantly decreasing AM counts (45). The intermediate role of the bone marrow means that the gut–lung axis pathway is highly collaborative, so it is probably feasible to retain the necessary antiinfection ability and achieve the anti-inflammatory ability of AMs. Moreover, although with no research on the role of GM in viral pneumonia, considering its previous description in modulating bacterial clearance, it stands to reason that the GM also has a function here, the altered hematopoietic function is likely not the sole source to prevent tissue damage. The supplementation of *Clostridium butyricum* can prevent excessive inflammation during RSV infection, probably because of the M2 polarization of AMs. Oral administration of *Lactobacillus rhamnosus* CRL 1505 activates AMs and enhances their production of type I interferon and interferon-γ as well as their ability to combat RSV. The enhanced antiviral response of AMs may be attributed to the increase of circulating SCFAs (46–48). These findings suggested that probiotics may be promising for preventing and treating viral infections; however, additional clinical studies are needed to further delineate their effects.

### 2.2 Inflammatory injury

AMs are the most abundant innate immune cell during acute lung injury (ALI) and the critical drivers of the inflammatory response (49). In elderly patients with ALI, changes in GM contribute to lung inflammation, which aggravates symptoms; this senescence-related susceptibility maybe due to AM aging (50). Intestinal ischemia/reperfusion (I/R) increases an individual’s susceptibility to ALI-related complications (51). Succinate, a metabolic byproduct of GM, accumulates during intestinal I/R and promotes lung damage by regulating AM polarization. Additionally, C5a produced during ALI promotes AM autophagy, leading to AM apoptosis and disruption in pulmonary homeostasis (52, 53). However, some treatment approaches play a protective role against ALI. The oral administration of propionic acid, another GM-derived metabolite, contributes to the suppression of AM-mediated inflammation during ALI in metal fume fever (54). Pretreatment with astragalus polysaccharides, a well-known immunoregulatory bioactive component, regulates AMs by changing the GM composition and increasing the SCFA level, which constitutes anti-inflammatory activity in ALI and plays a prophylactic role (55). Surprisingly, although MV is considered a routine and effective strategy to treat ALI, it probably increases gut permeability and enhances AM activation, which can exacerbate ALI and even cause systemic sepsis (56).

Chronic obstructive pulmonary disease (COPD) is a progressive disease with a significant incidence and mortality. Drug therapy can temporarily improve but not normalize lung function (57). Even after smoking cessation, other nondrug measures are necessary. AMs always remain activated in patients with COPD, aiding the characterization of the alveolar destruction that occurs during emphysema (58). The optimal depletion of potentially harmful bacteria and/or enrichment of beneficial bacteria in the gut may be alternative strategies for improving COPD. HFD can reduce the development of emphysema by inhibiting local and systemic inflammation. In previous studies, a significant decrease in the number of macrophages in bronchoalveolar lavage fluid was observed, as was a significant decrease in tissue destruction and the expression of healing-related factors that are mainly secreted by macrophages (59, 60). AMs play an important role in the response to pathogen infection in COPD lungs; however, additional studies should elucidate how to avoid damage to alveolar structures by strictly balancing AM activation.

Patients with chronic Inflammatory bowel disease (IBD) are more susceptible to developing chronic inflammatory lung diseases. In the absence of obvious lung disease, the lung function of IBD patients still worsens (61). Previous studies have reported that the lung tissue injury was accompanied by a significant increase of AMs in bronchoalveolar lavage fluid, while the increased expression of nicotinamide adenine dinucleotide phosphate oxidase 2 and C-C motif chemokine ligand 2 showed that AMs actively participated in the lung injury of patients with colitis (62). Thus, one may infer that GM disorder is an important driver for IBD complicated with chronic lung inflammation, as it is also a widely accepted important factor affecting AM functions during lung injury. In addition, colitis can affect bone marrow hematopoiesis, thereby enhancing monocyte release into the blood and infiltration into the lungs (63). Thus, monocyte-derived alveolar macrophages (Mo-AMs) may be an important driving factor of pulmonary inflammation in the context of colitis that is accomplished through a mechanism of the gut–bone marrow–lung axis, similar to our previous description of influenza infection and further extending that “double-edged benefit” concept.

Idiopathic pulmonary fibrosis (IPF) is a progressive and fatal lung disease with limited treatment options and a huge demand for new therapies (64). Through inhibiting histone deacetylase, a promising therapeutic target for IPF, enterogenous butyric acid promotes the expression of antifibrosis factors to ameliorate IPF, probably because butyric acid regulates the imbalance of macrophage subpopulations by affecting the differentiation of macrophages to AMs (65, 66). As determined earlier, Mo-AMs, rather than TRAMs, are the main driving factor for the development of pulmonary fibrosis, and the selective targeting and regulation of AM differentiation can improve fibrosis without adverse consequences related to the depletion of whole monocytes or tissue-intrinsic AMs. It is worth noting that, after the fibrosis subsides, the Mo-AMs continue to exist in the lung for 1 year, indicating the need for continuous targeted therapy (67).

Pulmonary arterial hypertension (PAH) is characterized by occlusive pulmonary vascular remodeling, and the pulmonary inflammation mediated by macrophages around the pulmonary vessels is a key driver leading to pathological changes occurring within it (68). No effective treatment is available, but the gut–lung axis was recently highlighted in these patients. Patients with PAH tend to have proinflammatory gut dysbiosis, and HFD may help slow the disease progression, probably because metabolite butyric acid reduces hypoxia-induced AM accumulation. During hypoxia-induced PAH, AM activation is complex and partially dependent on the microenvironment; thus, in addition to directly affecting the number of AMs, butyrate has a protective effect on hypoxia-induced angiogenesis and endothelial barrier dysfunction (69, 70). Besides, structural changes in the lungs are rarely reversible because PAH regression takes more time than PAH development. Therefore, butyrate may be more effective at preventing than reversing PAH symptoms.

### 2.3 Allergic diseases

Macrophages were once called “forgotten cells in asthma.” Asthma-related alveolar dysfunction can cause serious respiratory diseases, including AM death and surfactant dysfunction (71). The replacement of TRAMs by Mo-AMs in the alveolar ecological niche promotes the transition to hyperreactivity (5).

The increased fungal burden in the gut is associated with the risk of severe asthma (72). Through Abx-induced GM imbalance and fungal overgrowth, AMs are polarized into M2 macrophages and airway allergic inflammation is promoted. Correspondingly, the adoptive transfer of AMs to naïve objects can induce similar phenomena (73). Conversely, the presence of normal microbiota is conducive to inducing innate immune tolerance in the lung (74).

Epidemiological studies suggest that, as the fiber content in the diet increases, the incidence of asthma decreases (75). This is likely because HFD produces higher levels of SCFAs compared to a low-fiber diet, thereby reducing M2 polarization of AMs and better control of allergic inflammation. In a prospective cohort study, an acute soluble fiber challenge had an acute antiinflammatory effect on the asthmatic airway. It was observed that the sputum sample macrophage counts decreased after 4 hours. In summary, these studies suggest that AMs, as target cells for the gut–lung axis, may play an important role in regulating allergic pulmonary inflammation. In terms of mechanism, SCFAs regulate AMs by activating GPR43 and/or GPR41; interestingly, their effects on both receptors are contradictory to each other (76–78). However, the relationship between GPR41/GPR43 activation and AM differentiation remains unclear, and there are many possible explanations. Both receptors can be expressed in different cell subpopulations or tissues, thereby affecting different aspects of the immune response. The binding effect of GPR41/GPR43 receptors may be closely related to coreceptor or specific inflammatory cell differentiation, and further research must analyze the pleiotropic effects of GPR41/GPR43 (79, 80). It is worth noting that allergic inflammation usually exists long term and the TRAMs induced by the asthma response are replaced by Mo–AMs for at least 2 months after the allergy subsides (71). Most studies to date have focused only on the short-term benefits of HFD; thus, we must test whether a long-term HFD can promote the maintenance of the antiinflammatory phenotype of AMs. Moreover, AMs are associated with airway remodeling and airway hyperresponsiveness in chronic asthma (81, 82). Larger samples and longer clinical trials are necessary to determine the clinical effects of long-term supplementation.

### 2.4 Malignancy

Research is lacking on the effect of regulating AMs through the gut–lung axis on the occurrence and development of lung malignancies. AMs are widely involved in the formation of the tumor microenvironment, affecting tumor immunity and the immunotherapeutic response, which are important targets for cancer treatment (83, 84).

It has been reported that Influenza A virus infection allowing more effectively preventive antitumor immunity of mucosal resident AMs. This immune training strategy is particularly attractive to patients with extrapulmonary malignancies (such as melanoma and breast cancer) because they tend to develop lung metastases as well as for high-risk people with primary lung cancer. However, this kind of training immunity of AMs lasts for a limited time only (85). Maintaining sufficient long-term immune monitoring would be more beneficial for them, which illustrates the importance of continuous triggering factors. Nevertheless, it remains a promising approach to strengthening lung antitumor immunity.

Through long-term healthy dietary management, the risk of a new cancer diagnosis or cancer recurrence can be minimized, but most cancer patients struggle to maintain a beneficial and evidence-based diet after receiving the diagnosis, which may be due to differences in patient treatment and disease trajectory (86, 87). Providing appropriate dietary education is necessary; more importantly, understanding the relationship between diet and AM-mediated immunity is conducive to the development of personalized and practical nutritional plans. However, evidence of dietary interventions during tumor treatment is currently very limited (88). Via the gut–lung axis, it is unclear whether the anti-tumor function of AMs can be sustained. Additional research is urgently needed in this field. This treatment pattern, which focuses on the gut–distal organ axis–based regulation of tissue macrophages in the treatment of distal solid malignancy, not only provides new treatment targets and strategies to prevent and treat lung cancer, but it also provides new insights into the biological mechanism of systemic malignancy.

## 3 Conclusion

To our knowledge, the gut–lung axis is active throughout most of human life that has a continuous impact on AM function, origin, and renewal. A more thorough understanding of this multifaceted modulation has far-reaching significance. Dietary intervention, as we previously stated, has been widely studied for regulating AMs through the gut–lung axis (89). Dietary management is economical and universal, making it a feasible way to continuously trigger AMs to fight lung diseases. From above studies, dietary fiber can be transformed to prebiotics by probiotics, high-diet fiber diet tends to be beneficial in lung health by modulating proper AM immune response, but the effect of oral prebiotics is inconsistent, understanding their mechanism of action is conducive to the development of personalized and precision therapy. Moreover, although the concept that the gut–lung axis is a highly collaborative pathway based on AM plasticity is still in its infancy, its realization will make it feasible to develop personalized treatment with a double-edged benefit. However, the gut-lung axis is the mechanism with cross-organ communication, its further research on broader mechanisms are tough, but the application of induced pluripotent stem cell and multiorgan-on-a-chip technology have made it easier *in vitro*.

## Author contributions

ZC: Conceptualization, Data curation, Formal Analysis, Methodology, Writing – original draft, Writing – review & editing. YL: Data curation, Formal Analysis, Writing – review & editing. WH: Conceptualization, Project administration, Supervision, Writing – review & editing.
